# Study on the interaction of paeoniflorin with human serum albumin (HSA) by spectroscopic and molecular docking techniques

**DOI:** 10.1186/s13065-017-0348-3

**Published:** 2017-11-17

**Authors:** Liang Xu, Yan-Xi Hu, Yan-Cheng Li, Yu-Feng Liu, Li Zhang, Hai-Xin Ai, Hong-Sheng Liu

**Affiliations:** 1 College of Pharmacy, Liaoning University, Shenyang, 110036 People’s Republic of China; 2 Natural Products Pharmaceutical Engineering Technology Research Center of Liaoning Province, Shenyang, 110036 People’s Republic of China; 3 School of Life Science, Liaoning University, Shenyang, 110036 People’s Republic of China; 4 Research Center for Computer Simulating and Information Processing of Bio-macromolecules of Liaoning Province, Shenyang, 110036 People’s Republic of China; 5 Liaoning Engineering Laboratory for Molecular Simulation and Designing of Drug Molecules, Shenyang, 110036 People’s Republic of China

**Keywords:** Paeoniflorin, Human serum albumin, Fluorescence quenching, Molecular docking

## Abstract

The interaction of paeoniflorin with human serum albumin (HSA) was investigated using fluorescence, UV–vis absorption, circular dichroism (CD) spectra and molecular docking techniques under simulative physiological conditions. The results clarified that the fluorescence quenching of HSA by paeoniflorin was a static quenching process and energy transfer as a result of a newly formed complex (1:1). Paeoniflorin spontaneously bound to HSA in site I (subdomain IIA), which was primarily driven by hydrophobic forces and hydrogen bonds (ΔH° = − 9.98 kJ mol^−1^, ΔS° = 28.18 J mol^−1^ K^−1^). The binding constant was calculated to be 1.909 × 10^3^ L mol^−1^ at 288 K and it decreased with the increase of the temperature. The binding distance was estimated to be 1.74 nm at 288 K, showing the occurrence of fluorescence energy transfer. The results of CD and three-dimensional fluorescence spectra showed that paeoniflorin induced the conformational changes of HSA. Meanwhile, the study of molecular docking also indicated that paeoniflorin could bind to the site I of HSA mainly by hydrophobic and hydrogen bond interactions.

## Introduction

**Fig. 1:**
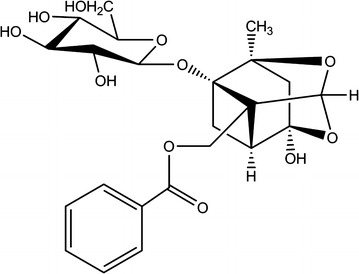
The structure of paeoniflorin

Radix Paeoniae Rubra (RPR), the dried root of *Paeonia lactiflora* Pall or *Paeonia veitchii* Lynch, has been widely used by Chinese medicine practitioners to treat cardiovascular, inflammation and female reproductive diseases [1]. Based on the principle of Chinese medicine, historical literatures described RPR with the functions of tonifying blood, cooling blood, cleansing heat and invigorating blood circulation [2]. The most abundant and active components in RPR are identified as paeoniflorin (PF) [3, 4] (C_23_H_28_O_11_, Fig. 1), which is reported to have many biological properties including antipyretic, antiallergic, antioxidative, antiinflammatory, and anxiolytic activities [5–7].

Protein is an important chemical substance in our life and one of the main targets of all medicines in organism. Human serum albumin (HSA) is the most studied serum albumin because its primary structure is well known and it can interact with many endogenous and exogenous substances [8]. It is a single-chain, non-glycosylated globular protein consisting of 585 amino acid residues, and 17 disulfide bridges assist in maintaining its familiar heart-like shape [9]. Crystallographic data show that HSA contains three homologous a-helical domains (I, II, and III): I (residues 1–195), II (196–383), and III (384–585), each of which includes 10 helices that are divided into six-helix and four-helix subdomains (A and B) [9]. The principal regions of ligand binding sites in HSA are located in hydrophobic cavities in subdomains IIA and IIIA, called site I and site II, respectively [10]. These multiple binding sites underline the exceptional ability of HSA to act as a major depot and transport protein which is capable of binding, transporting and delivering an extraordinarily diverse range of endogenous and exogenous compounds in the bloodstream to their target organs [11]. The binding affinity between serum albumin and many bioactive compounds is closely linked with the distribution and metabolism of these active ingredients [12–14]. Therefore, investigation of the binding of drug to HSA is of great importance to understand its effect on protein function during the blood transportation process and its biological activity in vivo.

HSA and BSA, two of the most extensively studied serum albumins, are homologous proteins. However, there are still some differences between them [15]. HSA contains a single tryptophan (Trp-214) [9], while BSA has two tryptophan residues that possess intrinsic fluorescence: Trp-212 is located within a hydrophobic binding pocket of the protein and Trp-134 is located on the surface of the molecule [16]. Therefore, the experimental results of the interaction between drugs and BSA cannot be completely identical with those of HSA. Although some spectroscopic studies on the interaction between paeoniflorin and bovine serum albumin (BSA) have been published [17–20], to our knowledge, a series of accurate and full basic data for clarifying the binding mechanisms of paeoniflorin to HSA remain unclear. Consequently, the binding characteristics of paeoniflorin with HSA including the quenching mechanism, quenching and binding constants were investigated in this study, by using fluorescence quenching method through the thermodynamic analysis. In addition, the conformational changes of HSA induced by paeoniflorin were also investigated by means of circular dichroism (CD) and three-dimensional fluorescence measurements. Finally, paeoniflorin molecule has been docked into the 3D structure of HSA in order to envisage a connection between the experimental and theoretical results. By comparing our results with those of previous studies, we can investigate the similarities and differences between paeoniflorin and two kinds of serum albumin.

## Experimental

### Materials

Commercially prepared human serum albumin (HSA, purity > 99.0%) was purchased from Sigma-Aldrich Co. (USA), and stored in refrigerator at 4.0 °C. Paeoniflorin, ibuprofen and warfarin were purchased from the National Institute for the Control of Pharmaceutical and Products (China). Samples were weighed accurately on a microbalance (Sartorius BP211D, Germany) with a resolution of 0.01 mg. The stock solutions of paeoniflorin, warfarin and ibuprofen (each 1.25 × 10^−3^ mol L^−1^) were prepared with 0.05 mol L^−1^ Tris–HCl buffer containing NaCl (0.05 mol L^−1^, pH 7.4). The HSA stock solution was dissolved and diluted to 1.0 × 10^−5^ mol L^−1^ with the same buffer, then was stored in the dark at 4 °C before fluorescence and UV–vis absorption essay. In the analysis of CD spectra, HSA stock solution (1.0 × 10^−6^ mol L^−1^) was prepared with phosphate buffer (0.05 mol L^−1^, pH 7.4). All other reagents were all of analytical reagent grade and were used as purchased without further purification. Double distilled water was used for all solution preparation.

### Methods

#### Fluorescence spectra

All the fluorescence spectra were carried out on an F-7000 fluorescence spectrophotometer (Hitachi High-technologies Co., Japan) equipped with a thermostatic bath. The fluorescence measurements were performed at three temperatures (288, 298, 310 K) in the range of 200–700 nm. The concentration of HSA was fixed at 1.0 × 10^−5^ mol L^−1^ and the concentrations of paeoniflorin changed from 0 to 1.25 × 10^−5^ mol L^−1^ at 2.5 × 10^−6^ mol L^−1^ intervals. The excitation and emission slit widths were both set at 5 nm. An excitation wavelength of 280 nm was set and the temperature of samples was maintained by recycling water during the whole experiment. All fluorescence titration experiments were done manually by the 25 μL microsyringe [21, 22]. In this work, the absorption wavelength of paeoniflorin was overlapped with the absorption wavelength of HSA. Thus, the fluorescence intensities of all HSA solutions were corrected for the inner-filter effect of fluorescence according to the following equation [23, 24]:

$${\text{F}}_{\text{corr}} = {\text{ F}}_{\text{obs}} \times {\text{e }}\left( {{\text{A}}_{\text{ex}} + {\text{ A}}_{\text{em}} } \right)/2$$

where F_corr_ and F_obs_ are the fluorescence intensity corrected and observed at the emission wavelength, respectively. A_ex_ and A_em_ are the absorbance of HSA at the excitation and emission wavelengths, respectively.

#### UV–vis absorption spectra

The UV–vis absorption spectra were recorded on a UV-2550 spectrophotometer (Shimadzu Co., Japan) over a wavelength range of 200–700 nm in a pH 7.4 Tris–HCl buffer at 298 K. Spectra of free paeoniflorin and paeoniflorin with 2.5 mL HSA solution were both measured. The concentrations of paeoniflorin varied from 0 to 5.0 × 10^−5^ mol L^−1^ at 1.0 × 10^−5^ mol L^−1^ intervals.

#### Binding competitive experiment

Two classical site probes, warfarin and ibuprofen, were selected as the markers of site I and site II separately. The concentrations of HSA and paeoniflorin were both fixed at 1.0 × 10^−5^ mol L^−1^, while the concentrations of the probes varied from 0 to 2.5 × 10^−5^ mol L^−1^ at 5.0 × 10^−6^ mol L^−1^ intervals. The experiment was carried out at room temperature. The wavelength range and the excitation wavelength remained unchanged [25].

#### Circular dichroism (CD) spectra

The CD spectra were measured on a J-810 automatic recording spectropolarimeter (Jasco Co., Japan) in the spectral range 200–240 nm under constant nitrogen flush. The solutions of HSA (1.0 × 10^−6^ mol L^−1^) and paeoniflorin (2.5 × 10^−5^ mol L^−1^) were both prepared with phosphate buffer.

#### Molecular docking

The molecular docking studies were performed to explore the interaction between paeoniflorin and HSA by using AutoDock program version 4.2.5.1 and AutoDockTools version 1.5.6, which is the graphical user interface of AutoDock supplied by MGL Tools [26]. The 3D structure of ligand (paeoniflorin) was constructed by ChemDraw. The default root, rotatable bonds and torsions of the ligand were set by AutoDockTools. The crystal structure of the Human Serum Albumin (PDB ID: 1AO6) was downloaded from the protein data bank (http://www.rcsb.org/pdb). All bound waters were removed from the protein using Pymol version 1.8.2.0. Polar hydrogen atoms were added, and AutoDock 4 atom types and Geisteger charges were assigned to the receptor protein using AutoDockTools. The docking site for the ligands on HSA was defined at the active site with grid box size of 60 × 60 × 60, spacing of 0.375 Å, and grid centre of 33.175, 30.604, and 34.136. The AutoGrid4 utility in AutoDock program was used to calculate the electrostatic map and atomic interaction maps for all atom types of the ligand molecule. The Lamarckian Genetic Algorithm (LGA) was selected with the population size of 150 individuals and with a maximum number of generations and energy evaluations of 27,000 and 2.5 million, respectively. During the docking procedure, the ligand was treated as flexible molecule and the receptor was kept rigid. Finally, 100 possible binding conformations were generated by AutoDock run. The best confirmation with least binding energy was visualized and analyzed by using PyMOl version 1.8.2.0 and Ligplot^+^ version 1.4.5 [27].

## Results and discussion

### Binding interaction of paeoniflorin with HSA

#### Quenching mechanism

**Fig. 2:**
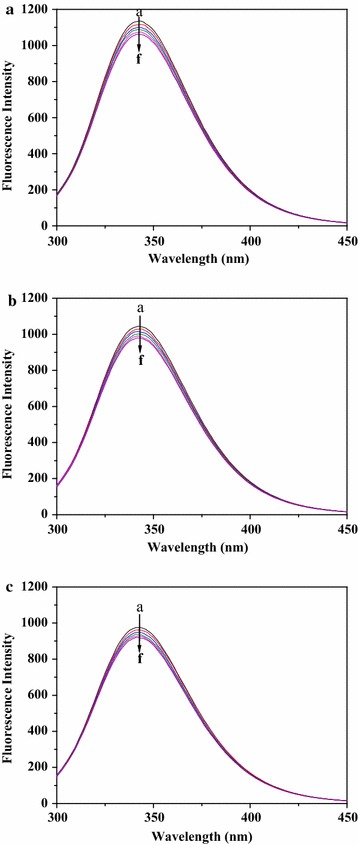
Fluorescence spectra of HSA + paeoniflorin solutions with paeoniflorin concentrations (a–f) (from 0.0 × 10^−5^ to 1.25 × 10^−5^ mol L^−1^ at 2.5 × 10^−6^ mol L^−1^ intervals) ([HSA] = 1.0 × 10^−5^ mol L^−1^, T = 288 K (**a**); 298 K (**b**); 310 K (**c**))

It has been reported that the tryptophan, tyrosine and phenylalanine residues give rise to the fluorescence of HSA [28]. As seen in Fig. 2, the emission of HSA was found to decrease progressively with increasing concentrations of paeoniflorin, showing that HSA had interacted with paeoniflorin.

Fluorescence quenching is usually classified into two types: dynamic quenching and static quenching. It can be distinguished by their different dependence on temperature and excited-state lifetime [23, 29]. For the dynamic quenching, higher temperatures will result in faster diffusion and larger amounts of collisional quenching. Therefore the quenching constant values will go up with the increase in temperature, but the reversed effect will be observed for static quenching [30]. To analyze the fluorescence quenching mechanism, the Stern–Volmer equation [31] was used:

$${\text{F}}_{0} /{\text{F }} = 1 + {\text{ K}}_{\text{SV}} \left[ {\text{Q}} \right] = 1 + {\text{ K}}_{\text{q}}\uptau_{0} \left[ {\text{Q}} \right]$$

F_0_ and F represent the fluorescence intensities of paeoniflorin in the absence and presence of the quencher, respectively. [Q] denotes the concentration of the quencher. K_SV_, K_q_, τ_0_ are the Stern–Volmer dynamic quenching constant, the quenching rate constant of the biomolecule (K_q_ = K_SV_/τ_0_), and the average lifetime of the fluorophore in the absence of quencher (τ_0_ = 6.0×10^−9^ s) [32], orderly.

**Fig. 3:**
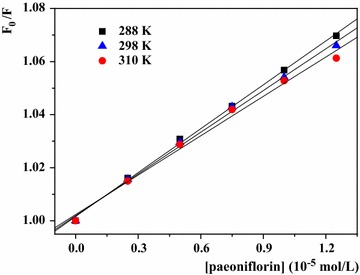
Stern–Volmer plots of HSA + paeoniflorin solutions with paeoniflorin concentrations (from 0.0 × 10^−5^ to 1.25 × 10^−5^ mol L^−1^ at 2.5 × 10^−6^ mol L^−1^ intervals) at three temperatures ([HSA] = 1.0 × 10^−5^ mol L^−1^)

As it was presented in Fig. 3 and Table 1, all of the three plots showed good linear relationship and the dynamic quenching rate constant was larger than the limiting diffusion constant of the biomolecule (2.0 × 10^10^ L mol^−1^ s^−1^) [33]. All of the above in this part declared that the quenching mechanism was static quenching.

**Table 1:** Quenching constants (K_SV_ and K_q_), stability constants (K_a_), correlation coefficients (R) and binding site numbers (n) and thermodynamic parameters calculated according to Stern–Volmer plots and double logarithm plots of HSA + paeoniflorin system at three temperatures

HSA + paeoniflorin (K)	K_SV_ (L mol^−1^)	K_q_ (L mol^−1^ s^−1^)	R^2^	K_A_ (L mol^−1^)	n	∆G^0^ (kJ mol^−1^)	∆H^0^ (kJ mol^−1^)	∆S^0^ (J mol^−1^ K^−1^)
288	0.569 × 10^4^	0.9483 × 10^12^	0.9965	1.909 × 10^3^	0.9053	− 18.10		
298	0.545 × 10^4^	0.9083 × 10^12^	0.9941	1.680 × 10^3^	0.8977	− 18.38	− 9.98	28.18
310	0.521 × 10^4^	0.8683 × 10^12^	0.9873	1.421 × 10^3^	0.8868	− 18.72		

From 0.00 × 10^−5^ to 1.25 × 10^−5^ mol L^−1^ at 2.50 × 10^−6^ mol L^−1^ intervals ([HSA] = 1.0 × 10^−5^ mol L^−1^, T = 288, 298 and 310 K)

**Fig. 4:**
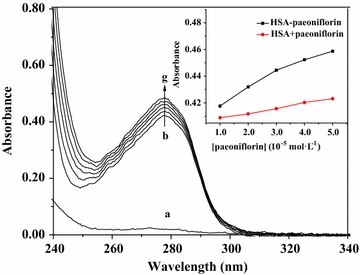
Absorption spectra of paeoniflorin alone (a) and HSA in the presence of different concentrations of paeoniflorin (b–g); Inset: comparison of the absorption values at 280 nm between the HSA–paeoniflorin mixed solutions and the sum values of free HSA and free paeoniflorin, a: [paeoniflorin] = 1.0 × 10^−5^ mol L^−1^; b–g: [HSA] = 1.0 × 10^−5^ mol L^−1^, [paeoniflorin] = 0, 1.0, 2.0, 3.0, 4.0, 5.0 × 10^−5^ mol L^−1^

UV absorption measurement is a very simple method and applicable to explore the complex formation [34, 35]. To confirm the result of fluorescence spectra, the UV spectra of HSA with the absence and presence of paeoniflorin were performed (Fig. 4). It revealed that the absorption of paeoniflorin was weak and the peak intensity of HSA rose with the addition of paeoniflorin. In addition, the inset in Fig. 4 demonstrated that the absorption values of simply adding free HSA and free paeoniflorin were obviously lower than those of HSA–paeoniflorin mixed solutions with the increasing concentrations of paeoniflorin. These results indicated that there was an interaction between paeoniflorin and HSA and a protein–ligand complex with certain new structure was formed [36]. And the quenching mechanism was the same as that of with BSA [18].

#### Binding constants and the number of binding sites

To further elucidate the binding constants (K_a_) and the number of binding sites (n), the modified Stern–Volmer equation was used [37]:

$${ \lg }\left[ {\left( {{\text{F}}_{0} - {\text{ F}}} \right)/{\text{F}}} \right] = {\text{ lg K}}_{\text{a}} + {\text{ n lg }}\left[ {\text{Q}} \right]$$

where K_a_ and n represent the binding constant and the number of binding sites, respectively. The other parameters in the equation have the same meaning as the Stern–Volmer equation above.

A linear plot based on lg [(F_0_ − F)/F] versus lg [Q] is expected, and n and K_a_ can be estimated from the slope and intercept.

**Fig. 5:**
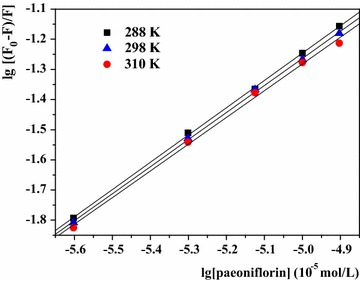
Double logarithm plot of HSA + paeoniflorin solutions with paeoniflorin concentrations (from 0.0 × 10^−5^ to 1.25 × 10^−5^ mol L^−1^ at 2.5 × 10^−6^ mol L^−1^ intervals) at three temperatures ([HSA] = 1.0 × 10^−5^ mol L^−1^)

The double logarithm plots at different temperatures were presented in Fig. 5 and the related statistics were listed in Table 1. The K_a_ values were in the order of 10^3^, revealed the binding of HSA–paeoniflorin complex was weak. The binding constant (K_a_) is especially significant to understand drug distribution in plasma. The drug like paeoniflorin with low binding constants of protein can improve the plasma concentrations of free drug, and then enhance its distribution and pharmacological effect [22]. Hence paeoniflorin usually has fast elimination and short maintenance time in vivo, which is in accordance with previous studies [38, 39]. In addition, it was clear that K_a_ declined as the temperature was on the rise, indicating that the stability of HSA–paeoniflorin complex decreased with the increasing temperature [40]. Besides, the number of binding sites approximated to 1. Thus, there was only one binding site between HSA and paeoniflorin which was the similar to BSA-paeoniflorin complex [18].

#### Thermodynamics of the HSA–paeoniflorin interactions

There are mainly four interaction forces between small molecules and biomolecules including Van der Waals forces, electrostatic forces, hydrogen bonds and hydrophobic interactions [28]. The thermodynamic parameters are important when determining the interaction force. The binding force was examined by Van’t Hoff equation:

$${\text{ln K}}_{\text{a}} = - \Delta {\text{H}}^\circ /{\text{RT }} + \Delta {\text{S}}^\circ /{\text{R}}$$

ΔH° and ΔS° are the enthalpy change and the entropy change, respectively, both of which can be evaluated from the slope and intercept of the linear plot of ln K_a_ against 1/T. K_a_ is the binding constant at different temperature. R and T represent the gas constant and temperature, respectively.

Obtaining the enthalpy change and the entropy change, the free energy change (ΔG°) can be calculated as well from the equation:

$$\Delta {\text{G}}^\circ = \Delta {\text{H}}^\circ - {\text{ T}}\Delta {\text{S}}^\circ$$

**Fig. 6:**
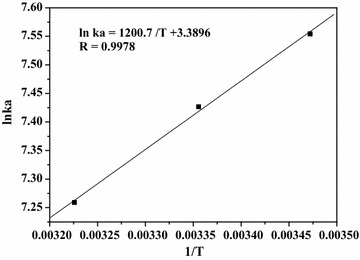
Van’t Hoff plot for the interaction of paeoniflorin with HSA with paeoniflorin concentrations (from 0.0 × 10^−5^ to 1.25 × 10^−5^ mol L^−1^ at 2.5 × 10^−6^ mol L^−1^ intervals) at three temperatures ([HSA] = 1.0 × 10^−5^ mol L^−1^)

As shown in Fig. 6 and Table 1, the free energy change (ΔG°) demonstrated the process of binding was spontaneous. Researchers [41] had concluded the rules of thermodynamics to determine the binding properties of biomolecules and small molecules. As the aqueous solution in the complex formation of paeoniflorin with HSA, the positive value of ΔS° (28.18 J mol^−1^ K^−1^) is regularly regarded as an evidence of hydrophobic interaction, because the water molecules that are arranged in an orderly way around the ligand and protein acquire a more random configuration [42]. Besides, the negative value of ΔH° (− 9.98 kJ mol^−1^) can be mainly attributed to hydrogen bonds since the structure of paeoniflorin consists of an ester group and several hydroxyl groups. Therefore, hydrophobic interactions and hydrogen bonds play major roles in the binding process and contribute to the stability of the paeoniflorin–HSA complex [36, 42]. It is obvious that the binding forces obtained in this study are more reasonable than that in Haiyan Wen et al’s work.

#### Binding site

**Fig. 7:**
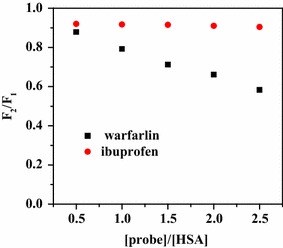
Effect of site maker probes on the fluorescence of HSA + paeoniflorin system ([HSA] = [paeoniflorin] = 1.0 × 10^−5^ mol L^−1^)

There are two main sub-domains of HSA namely sub-domains IIA and sub-domains IIIA which are the major ligand-binding sites: site I and site II [43]. To further detect the binding site of paeoniflorin with HSA, the competitive binding experiment was carried out. Warfarin and ibuprofen especially bound to site I and site II, respectively, were chosen as the site markers [23, 44]. According to the Fig. 7, the impact of warfarin on the fluorescence intensity was significant whereas there was almost no change caused by ibuprofen. With the increasing addition of warfarin, there was an obvious decline of the fluorescence intensity. Therefore, paeoniflorin shared a common binding site with warfarin, namely site I.

#### The energy transfer of paeoniflorin with HSA

**Fig. 8:**
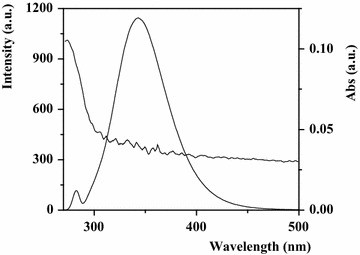
Spectral overlap of fluorescence of HSA solution and absorption of paeoniflorin solutions ([HSA] = [paeoniflorin] = 1.0 × 10^−5^ mol L^−1^, T = 288 K)

According to the Förster’s non-radioactive energy transfer theory, when there was an overlapping phenomenon between the emission peak of the donor (HSA) and the absorption peak of the acceptor (paeoniflorin) as shown in Fig. 8, fluorescence energy transfer would occur [45]. Depending on the equations of Förster resonance energy transfer as follows, the binding distance of the complex was worked out in Table 2.

**Table 2:** Energy transfer efficiency (E), critical binding distance (R), overlap integral (J) and binding distance (r) calculated according to Föster’s non-radioactive energy transfer theory

System	E (%)	R (nm)	J (cm^3^ L mol^−1^)	r (nm)
HSA + paeoniflorin	5.37	1.08	0.729 × 10^−16^	1.74

([HSA] = [paeoniflorin] = 1.00 × 10^−5^ mol L^−1^, T = 288 K)

The efficiency of energy transfer (E) was calculated by:

$${\text{E}} = 1 { }{-}{\text{ F}}/{\text{F}}_{0} = {\text{ R}}^{ 6} /\left( {{\text{R}}^{ 6} + {\text{ r}}^{ 6} } \right)$$

F and F_0_ indicate the fluorescence intensities of HSA in the presence and absence of paeoniflorin, respectively. R and r denote the critical binding distance and binding distance between HSA and drug.

The critical distance (R) was obtained by the following equation:

$${\text{R}}^{ 6} = 8.78\times 10^{-23} {\text{k}}^{ 2} {\text{N}}^{-4}\upphi{\text{J}}$$

where k^2^ stands for the dipole orientation factor; N is the refractive index of the medium; ϕ and J signify the fluorescence quantum yield of the donor and the overlap integral, separately.

The overlap integral was got from the equation:

$${\text{J }} = {{\sum {\text{F}}(\uplambda)\upvarepsilon(\uplambda)\uplambda^{ 4} \Delta\uplambda} /{\sum {\text{F}}(\uplambda)\Delta\uplambda}}$$

in which F(λ) represents the fluorescence intensity of the fluorescent donor at wavelength λ, and ε(λ) is the molar absorption coefficient of the acceptor at wavelength λ [46].

According to calculation, the values of E, R, J, r were 5.37%, 1.08 nm, 0.729 × 10^−16^ cm^3^ L mol^−1^ and 1.74 nm, respectively. The result of binding distance (r) below 8 nm and the fulfillment of the required condition 0.5 R < r < 2 R suggested that a high probability of the energy transfer occurred between paeoniflorin and HSA [47], which was reported for the first time.

### Conformation investigation

In general, the conformation of HSA will change when it is bound to small molecules. In this part, three-dimensional fluorescence spectra, CD spectra and molecular modeling were introduced to investigate it.

#### Three-dimensional (3D) fluorescence spectra

**Fig. 9:**
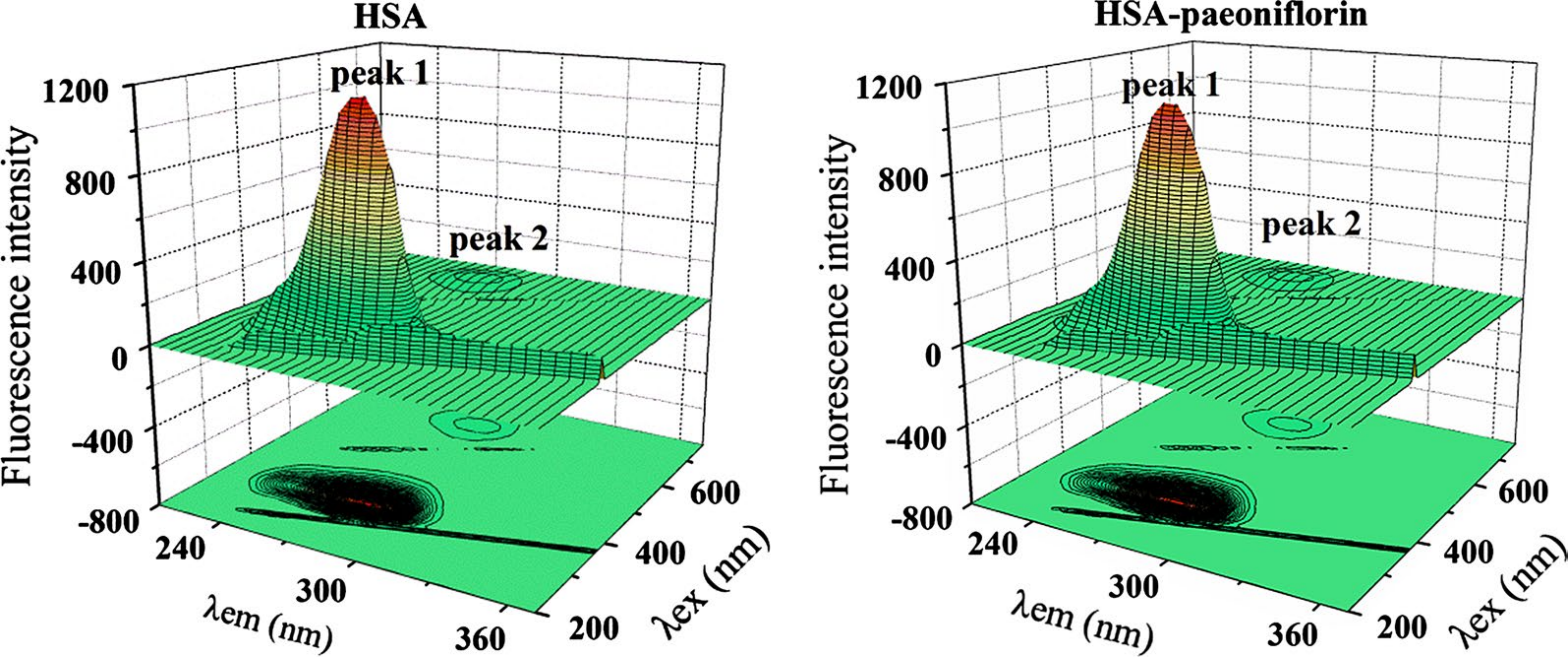
Three-dimensional fluorescence spectra and corresponding contour diagrams of free HSA, HSA + paeoniflorin systems ([HSA] = 1.0 × 10^−5^ mol L^−1^, [paeoniflorin] = 1.25 × 10^−5^ mol L^−1^)

Three-dimensional fluorescence spectra have gained growing popularity in detecting protein conformational changes that make the result more visual and credible [48, 49]. Both the three-dimensional spectra and the contour diagrams of HSA in the absence and presence of paeoniflorin were exhibited in Fig. 9. The corresponding characteristic parameters were shown in Table 3. In the 3D figures, peak 1 denoted the intrinsic fluorescence of tryptophan and tyrosine residues. Peak 2 revealed the spectral behavior of polypeptide backbone structures, and it was also connected with the change of secondary structure of HSA. According to the figure and the table, it was clear that there was a drop in fluorescence intensity of both peak 1 and 2 when paeoniflorin was added to HSA. Meanwhile, the addition of paeoniflorin caused blue shift (5 nm) of peak 1. It suggested that paeoniflorin interacted with HSA and led to the conformational change of the biomolecule [48].

**Table 3:** Three-dimensional fluorescence spectral characteristic parameters of free HSA system, HSA + paeoniflorin systems

System	Peak 1			Peak 2		
	Peak position λ_ex_/λ_em_ (nm/nm)	Strokes shift Δλ (nm)	Intensity	Peak position λ_ex_/λ_em_ (nm/nm)	Strokes shift Δλ (nm)	Intensity
Free HSA	280.0/345.0	65	1149	280.0/665.0	380	49.18
HSA + paeoniflorin	280.0/340.0	60	1112	280.0/670.0	390	47.07

#### CD spectra

**Fig. 10:**
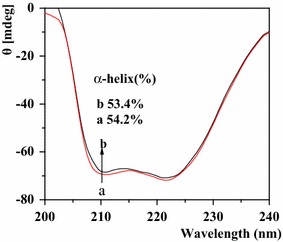
CD spectra of HSA in the absence (a) and presence of (b) paeoniflorin ([HSA] = 1.0×10^−6^ mol L^−1^, [paeoniflorin] = 2.5×10^−5^ mol L^−1^)

CD spectra is a sensitive method to identify the conformational changes of protein [50]. As seen from Fig. 10, there were two obvious negative bands of HSA in the ultraviolet region at 210, 222 nm that were the characteristic structure of *α*-helix of protein [51]. In the presence of paeoniflorin, the signal of CD decreased. Changes in α-helical content can be investigated by the peak decreasing or increasing and also be calculated by the following two equations:

$${\text{MRE }} = {\text{ observed CD }}\left( {\text{mdeg}} \right)/( 10 \times {\text{Cpnl}})$$

$$\upalpha{\text{-helix }}\left( \% \right) = \left[ {\left( { - {\text{MRE}}_{ 20 8} - 4000} \right)/\left( { 33000 - 4000} \right)} \right] \times 100$$

wherein MRE (mean residue ellipticity) is ellipse rate of the average residues; Cp is the mole fraction of protein; n is the number of amino acid residues; l is the light path of sample cell. According to the calculation result, the percentage of *α*-helix of HSA declined slightly from 54.2 to 53.4%, indicating that paeoniflorin induced a slight change of helical structure content of HSA [52, 53].

#### Molecular docking

The thermodynamics study illustrated that the main forces among the HSA–paeoniflorin complex were hydrophobic forces and hydrogen bonding which were not completely identical with Han-Yan Wen’s work [18]. Meanwhile, molecular docking was used to verify the theoretical calculations in this experiment.

**Fig. 11:**
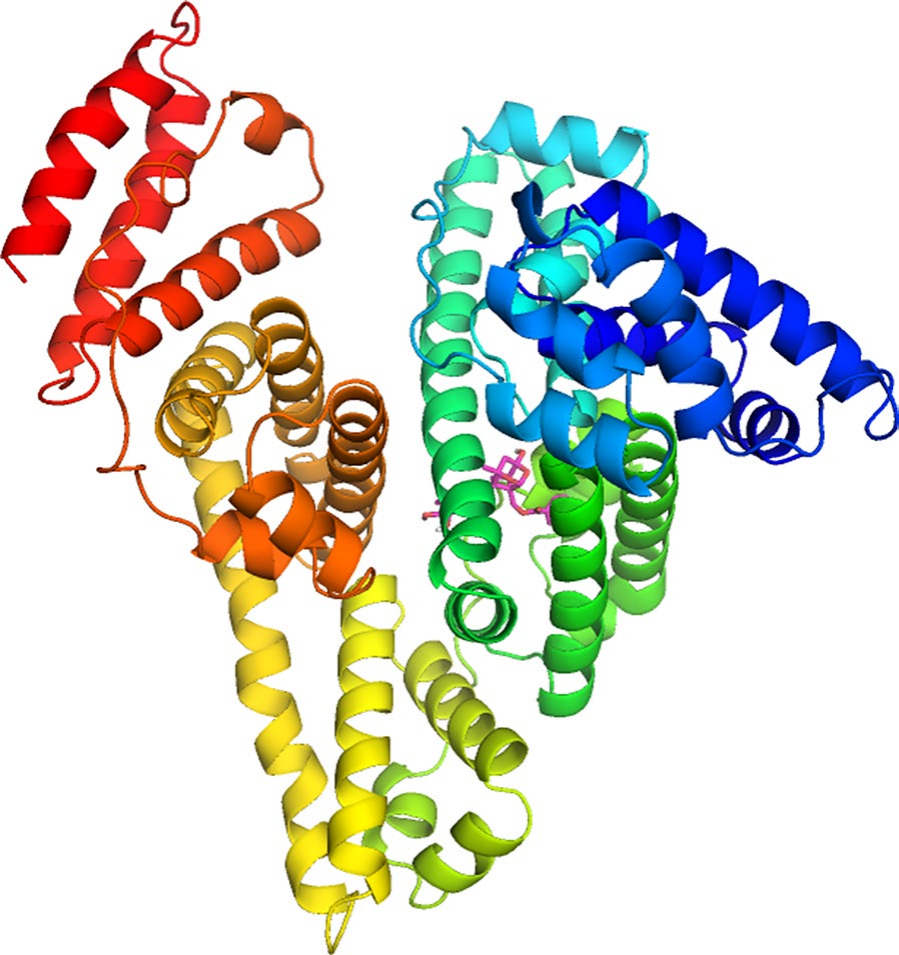
Paeoniflorin docked in the binding pocket of HSA

**Fig. 12:**
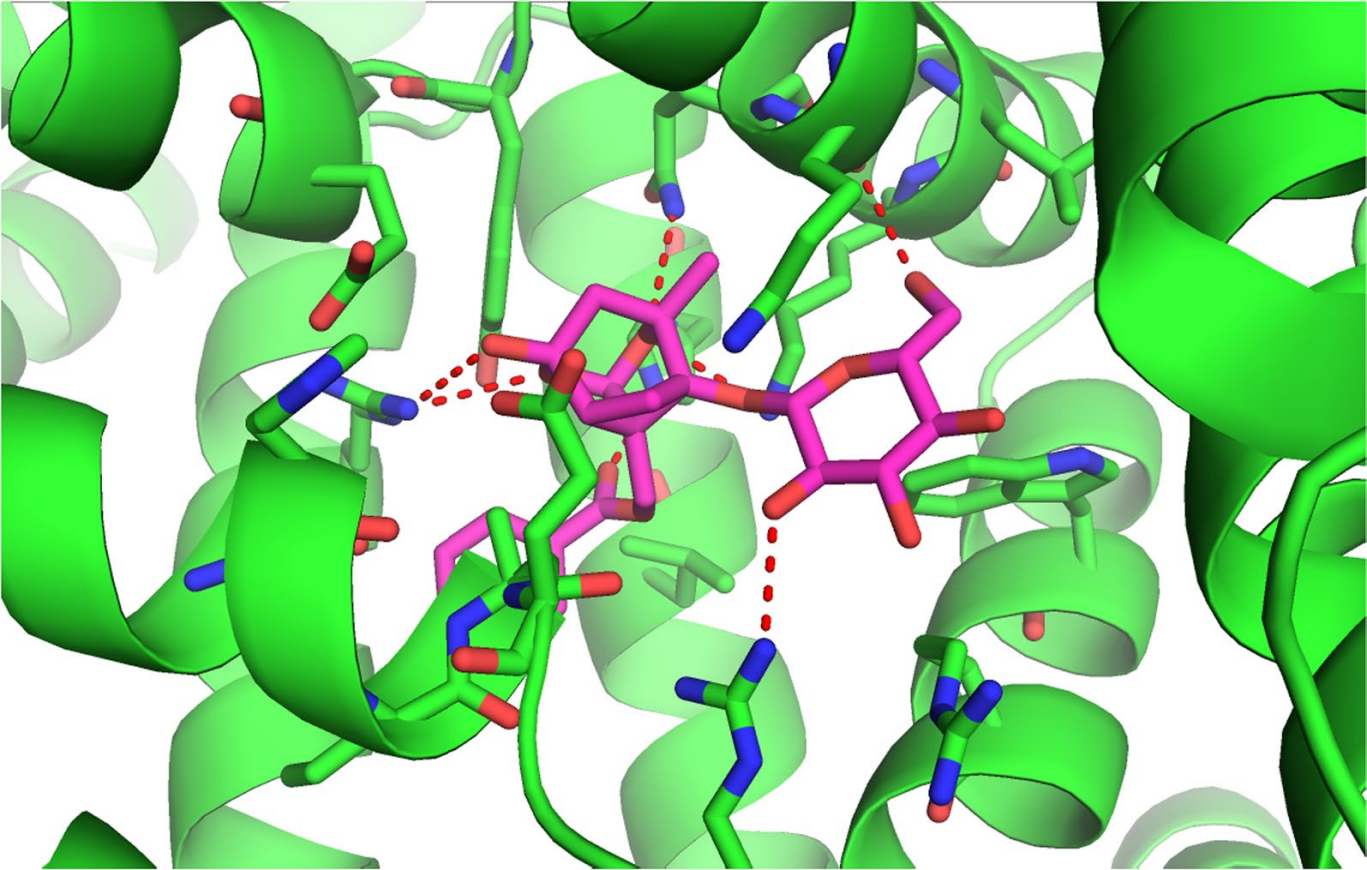
The active site residues of HSA and paeoniflorin. The HSA is presented by ribbon structure whereas paeoniflorin by stick model

**Fig. 13:**
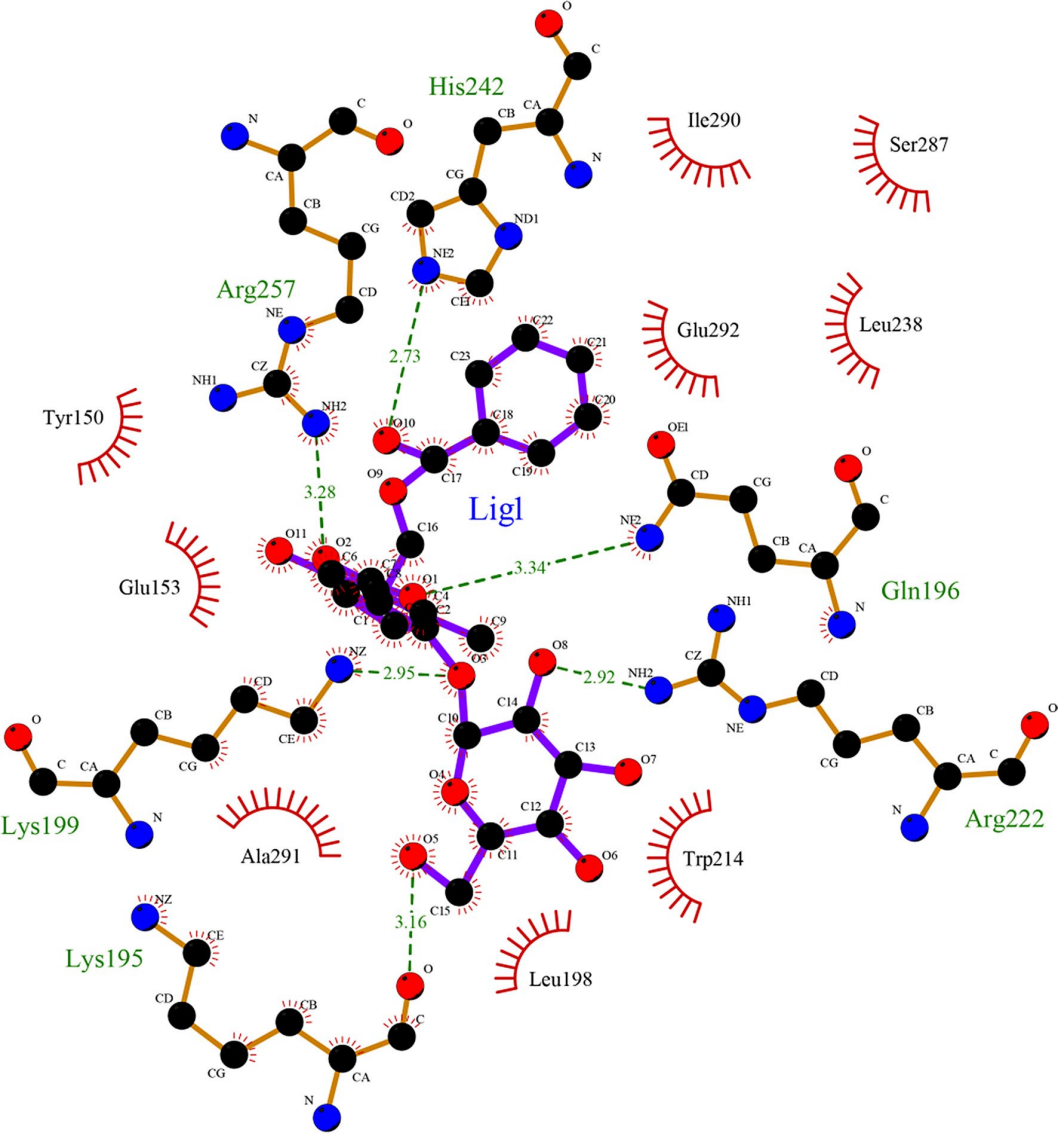
The interaction model of paeoniflorin at site I of HSA with its hydrogen bodings and hydrophobic interactions

Molecular docking, visually exhibiting the stereo binding modes, is increasingly used in the study of interaction between biomolecule and small molecules. The possible HSA–paeoniflorin binding mode was predicted by molecular docking software AutoDock. On the basis of the best binding confirmation, the molecular interactions were depicted below (Figs. 11, 12). This result confirmed that paeoniflorin bound into the sub-domain IIA of HSA, namely site I [44, 46, 52]. It revealed that Y150, E153, K195, Q196, L198, K199, W214, R218, R222, L238, H242, R257, S287, H288, I290, A291 and E292 of HSA interacted with paeoniflorin. In addition, according to the analysis of Ligplot^+^ (Fig. 13), K195, Q196, K199, R222, H242 and R257 of HSA combined paeoniflorin with hydrogen bonds and Y150, E153, A291, L198, W214, E292, L238, S287 and I290 bound paeoniflorin via hydrophobic forces, which has not been reported before. Based upon the molecular docking results, it was concluded that several amino acid residues played an important role in forming the binding of paeoniflorin and HSA. The molecular docking results indicated that the interaction between paeoniflorin and HSA was dominated by hydrophobic forces as well as hydrogen bonding, which were consistent with our experimental results.

## Conclusions

In this paper, the interaction of paeoniflorin with HSA was investigated by fluorescence, UV–vis, CD and molecular docking techniques under simulated physiological conditions. In addition, our results compared with previous work were also discussed. The results demonstrated that the fluorescence of HSA would be quenched with the addition of paeoniflorin. This change was via static quenching and energy transfer. According to Stern–Volmer equation, the binding constant was calculated (1.909 × 10^3^ L mol^−1^, 288 K). Besides, the study of thermodynamics parameters with negative value of ∆H°, ∆G°, and positive value of ∆S° indicated that the process was spontaneous and was mainly driven by hydrophobic interactions and hydrogen bonds. In accordance with the Förster’s non-radioactive energy transfer theory, the binding distance between paeoniflorin and HSA was evaluated as 1.74 nm. The results of the current study suggest that paeoniflorin can bind to HSA and form 1:1 complex. Analysis of molecular probes and molecular docking showed that the binding site located in Sudlow’s site I. Combined with paeoniflorin, the conformation of HSA changed according to the results of 3D, UV–vis and CD spectra. Additionally, paeoniflorin may induce conformational changes of HSA and affect its biological function as the carrier protein.

The conclusions are important in the field of pharmacology and biochemistry and are helpful for understanding the effect of paeoniflorin on protein function during the blood transportation process and its biological activity in vivo. The clear and quantitative information on the nature of paeoniflorin–HSA interaction may provide some information for its rational use in clinical practice.
